# Utility of *Plasmodium falciparum* DNA from rapid diagnostic test kits for molecular analysis and whole genome amplification

**DOI:** 10.1186/s12936-020-03259-9

**Published:** 2020-05-27

**Authors:** Suttipat Srisutham, Kanokon Suwannasin, Vivek Bhakta Mathema, Kanlaya Sriprawat, Frank M. Smithuis, Francois Nosten, Nicholas J. White, Arjen M. Dondorp, Mallika Imwong

**Affiliations:** 1 Department of Clinical Microscopy, Faculty of Allied Health Sciences, Chulalongkorn University, Bangkok, Thailand; 2 Mahidol-Oxford Tropical Medicine Research Unit, Faculty of Tropical Medicine, Mahidol University, Bangkok, Thailand; 3 Department of Molecular Tropical Medicine and Genetics, Faculty of Tropical Medicine, Mahidol University, Bangkok, Thailand; 4 Shoklo Malaria Research Unit, Faculty of Tropical Medicine, Mahidol University, Mae Sot, Thailand; 5 Myanmar Oxford Clinical Research Unit, Yangon, Myanmar; 6 Centre for Tropical Medicine and Global Health, Nuffield Department of Medicine, University of Oxford, Oxford, UK; 7 Medical Action Myanmar, Yangon, Myanmar

**Keywords:** *Plasmodium falciparum*, RDT, Whole genome amplification

## Abstract

**Background:**

Rapid diagnostic tests (RDTs) have become the most common diagnostic tool for detection of *Plasmodium falciparum* malaria, in particular in remote areas. RDT blood spots provide a source of parasite DNA for molecular analysis. In this study, the utility of RDTs for molecular analysis and the performance of different methods for whole genome amplification were investigated.

**Methods:**

Positive *P. falciparum* RDTs were collected from Kayin, Myanmar from August 2014 to January 2016. The RDT samples were stored for 6 months, 9 months, 20 months, 21 months, and 32 months before DNA extraction and subsequent molecular analysis of *P. falciparum kelch 13* (*pfkelch13*) mutations, *P. falciparum multidrug resistance 1* (*pfmdr1)*, and *P. falciparum plasmepsin 2* (*pfplasmepsin2*) gene amplification. In addition, performance of four whole genome amplification (WGA) kits were compared, including REPLI-g^®^, MALBAC^TM^, PicoPLEX^®^, and GenomePlex^®^, for which DNA quantity and quality were compared between original DNA and post-WGA products.

**Results:**

The proportion of successful amplification of the different molecular markers was similar between blood spots analysed from RDTs stored for 6, 9, 20, 21, or 32 months. Successful amplification was dependent on the molecular markers fragment length (*p value *< 0.05): 18% for a 1245 bp fragment of *pfkelch13*, 71% for 364 bp of *pfkelch13*, 81% for 87 bp of *pfmdr1*, 81% for 108 bp of *pfplasmepsin2*. Comparison of the four WGA assay kits showed that REPLI-g^®^, MALBAC^TM^, and PicoPLEX^®^ increased the quantity of DNA 60 to 750-fold, whereas the ratio of parasite DNA amplification over human DNA was most favourable for MALBAC^®^. Sequencing results of *pfkelch13*, *P. falciparum chloroquine resistance transporter* (*pfcrt*), *P. falciparum dihydrofolate reductase* (*pfdhfr*) and six microsatellite markers assessed from the post-WGA product was the same as from the original DNA.

**Conclusions:**

Blood spots from RDTs are a good source for molecular analysis of *P. falciparum*, even after storage up to 32 months. WGA of RDT-derived parasite DNA reliably increase DNA quantity with sufficient quality for molecular analysis of resistance markers.

## Background

Rapid diagnostic test kits (RDTs) have become the most common diagnostic tool for identifying patients with *Plasmodium falciparum* malaria, in particular in more remote settings where microscopy services are not readily available [1]. RDTs testing positive for *P. falciparum* are a potential source of *P. falciparum* DNA. Previous studies have described several techniques to recover *Plasmodium* DNA from RDT [2–4] and different DNA extraction methods vary regarding quality and quantity of obtained parasite DNA [2]. Out of three different extraction techniques evaluated, phenol/chloroform extraction was most efficient in obtaining parasite DNA from RDT samples [2]. RDT derived *Plasmodium* DNA has been evaluated for assessment of *18S rRNA* [2, 4, 5], the *P. falciparum histidine rich protein*-*2* and *3* genes [6], *P. falciparum* polymorphic microsatellite markers [7], drug resistance markers *pfcrt* and *pfmdr1* [8] and *tRNA methionine*-based quantification of *Plasmodium* parasites [9]. Successful assessment varied between 70% and 100% depending on the target gene and method. Previous study shows that the RDT sample stored at room temperature for 36 months (n = 1) was successfully amplified for *18S rRNA* gene based on nested PCR assay [2]. However, the limited quality and quantity of extracted DNA from RDT blood spots remains an important issue for its use in molecular genetic investigations.

Whole genome amplification (WGA) is a technique to increase the quantity of DNA including *Plasmodium* DNA from RDTs [10]. Several commercial kits are available for WGA, including REPLI-g^®^, MALBAC^TM^, PicoPLEX^®^, and GenomePlex^®^. The REPLI-g^®^ (Qiagen, Hilden, North Rhine-Westphalia, Germany) is based on a multiple displacement amplification (MDA) method utilizing phi29 DNA polymerase in isothermal amplification [11], and has been used successfully to amplify *P. falciparum* DNA from patient samples [12, 13]. MALBAC^TM^ (Yikion Genomic, Nantong, China) is based on multiple annealing and looping-based amplification cycles. Studies on human DNA showed that MALBAC^TM^ had very high amplification homogeneity, a low allele dropout rate, good reproducibility and adequate detection of copy number variations (CNVs) and single nucleotide polymorphisms (SNPs) [14]. PicoPLEX^®^ (Takara Bio, Mountain View, California, U.S.A.) and GenomePlex^®^ (Sigma–Aldrich, Foster City, California, U.S.A.) are based on degenerate-oligonucleotide-primed PCR (DOP-PCR). Comparisons between PicoPLEX^®^ and GenomePlex^®^ showed that PicoPLEX^®^ provided higher reproducibility and lower sequencing error frequency than GenomePlex^®^, whereas Genomplex had lower GC-bias than PicoPLEX^®^ [15]. The size of amplicons varied with different WGA kits: 4.0–10.0 kb in REPLI-g^®^, 0.5–2.0 kb in MALBAC^TM^, 0.25–1.0 kb in PicoPLEX^®^, and 0.1–1.0 kb in GenomePlex^®^ [16–18]. However, a comparative study of WGA kits focusing on the quality and quantity of *P. falciparum* DNA obtained from stored RDT samples has not been reported.

The current study assessed the utility for molecular marker analysis of DNA extracted from stored malaria RDTs and the effect of the duration of storage. In addition, four different WGA kits were evaluated for the quantitative and qualitative yield of parasite DNA form stored RDTs, including an assessing concordance of the molecular markers results between original DNA and post-WGA samples. Finally, estimated costs and time consumption were briefly discussed.

## Methods

Ethical approvals for the study were obtained from the ethical review committees of the Faculty of Tropical Medicine, Mahidol University (MUTM 2012-045-05) and the Department of Medical Research, Myanmar (Ethics/DMR/2015/109E and Ethics/DMR/2015/113E). Informed consent was obtained from all participants.

### RDT samples and DNA extraction

Used RDT kits SD BIOLINE Malaria Ag *P.f/P.v* kits (Abbott Diagnostics, USA) from confirmed *P. falciparum* positive cases (n = 108) were collected from Kayin State, Myanmar from August 2014 to January 2016, which obtained from the previous study reviewed and approved by the Ethical Review Committee of the Department of Medical Research, Myanmar (Ethics/DMR/2015/113E, Ethics/DMR/2015/109E). They were used for the evaluation of the impact of RDT samples stored for 6 months (n = 27), 9 months (n = 5), 20 months (n = 36), 21 months (n = 36), and 32 months (n = 4) at room temperature until DNA extraction and molecular analysis. Positive control samples from microscopically confirmed *P. falciparum* malaria patients from Northeastern Thailand (n = 5) were prepared by loading 5 µl of whole blood samples on the RDT, and stored for 24 h before DNA extraction and molecular analysis.In addition, confirmed *P. falciparum* positive RDT kits (n = 33) were used for validation of four WGA kits (n = 8) and analysis of the analytical concordance between the molecular marker results of original DNA and post-WGA samples (n = 25).

The RDT samples were extracted using the elution methods adapted from previous study [2, 3]. Crude DNA extraction was performed by cutting the filter paper in the RDTs, elute the DNA using PCR-grade water (100 µl), mixing by vortex, incubating at 95 °C for 10 min, and transferring the supernatant into a DNA collection tube.

### Utility of DNA from RDTs for molecular analysis

DNA samples extracted from RDTs were used to analyse the polymorphism of *P. falciparum* kelch-13 (*Pfkelch13*), *pfmdr1*, and *pfplasmepsin2* gene amplification. A total of 108 samples were analysed for *Pfkelch13* mutations based on PCR assays of 1245 bp and 364 bp, respectively. The samples were also analysed for *pfmdr1* (n = 108) and *pfplasmepsin2* (n = 108) CNVs based on relative quantitative real-time PCR assay as described previously for *pfmdr1* [19] and *pfplasmepsin2* [20]. The success rates for each molecular marker was calculated as:

$$ \left( {{100}\times\frac{{{\text{Number of samples successfully amplified}}}}{{{\text{Total of samples tested}}}}} \right). $$

Comparison of success rates between storage duration and between each molecular marker were determined using Chi Square Tests (SPSS version 25).

### Validation of four WGA kits

**Fig. 1:**
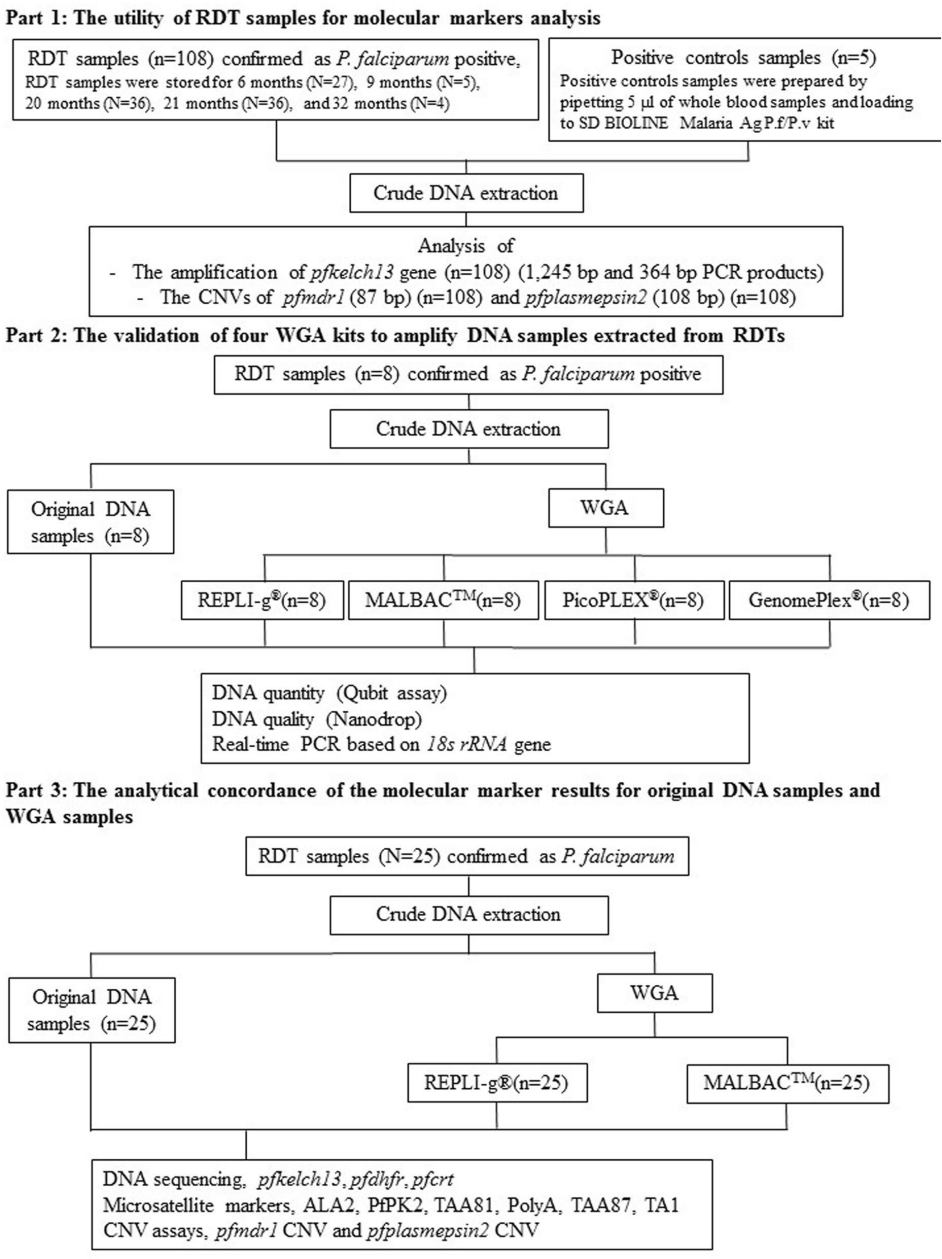
Schematic overview of the study design

DNA samples (n = 8) were extracted from RDTs kits and positivity for *P. falciparum* was confirmed using *18S rRNA* gene [21, 22]. Before and after whole genome amplification, the DNA samples were purified using DNA clean kit (Zymoresearch, USA). Five microliters of cleaned DNA samples (n = 8) were used following standard manufacturer protocols of the different WGA kits, including REPLI-g^®^ Midi Kits (Qiagen, Germany), MALBAC^TM^ WGA kit (Yikon Genomics, China), PicoPLEX^®^ (Takara Bio, U.S.A.) and GenomePlex^®^ (Sigma–Aldrich, U.S.A.) (Fig. 1). Human DNA samples without *P. falciparum* DNA was used as positive control for WGA and as negative control for parasites DNA amplification.

To assess the efficiency of whole genome amplification, original DNA samples extracted from RDT and post-WGA samples were checked for DNA quality using Nanodrop and DNA quantity using Qubit kit. To measure the quantity of parasites DNA, relative quantitative real-time PCR assay was performed based on amplification of the *18S rRNA* gene [21, 22]. The amplification performance of the kits was evaluated based on the quantitative increase in overall DNA, parasite DNA, and the ratio between parasite and human DNA in the amplified sample. Reproducibility of WGA was determined based on the coefficient of variation of the fold-change of DNA concentration for each WGA kit.

### Molecular markers analysis of original DNA and post-WGA samples

To evaluate the accuracy of molecular marker assessments from post-WGA samples compared to the original DNA sample. Crude DNA extracts from *P. falciparum* positive RDTs kits (n = 25) were purified using DNA clean kit, and then 5 µL of each purified DNA was subjected to amplification using both the REPLI-g^®^ and MALBAC^TM^ kits. DNA samples of original DNA and post-WGA were accessed using real-time PCR assay for molecular markers, such as *pfmdr1* [19] and *pfplasmepsin2* CNVs [20]. In addition, the amplicons of *pfkelch13* [23], *P. falciparum chloroquine resistance transporter* gene (*pfcrt*), *P. falciparum dihydrofolate reductase* gene (*pfdhfr*) and six micro-satellite markers (ARA2, PfPK2, TAA81, Poly A, TA1, and TAA87) [24] were sequence-analysed. The analytical concordance between the results obtained pre- and post-WGA were assessed using Kappa analysis (SPSS version 25). The success rate of molecular markers analysis of each WGA samples were calculated using the formula:

$$ \left( {\text{100}\,{ \times }\,\frac{{\text{Number}\,\text{of}\,\text{markers}\,\text{successfully}\,\text{amplified}}}{{\text{Total}\,\,\text{of}\,\text{markers}\,\text{tested}}}} \right)\text{.} $$

The association of molecular marker success rate and the initial parasites DNA concentration were determined using Spearman’s rank-order test using SPSS version 25.

## Results

### Success of *P. falciparum* gene amplification from DNA preps isolated from RDT samples upon varied period of storage

**Fig. 2:**
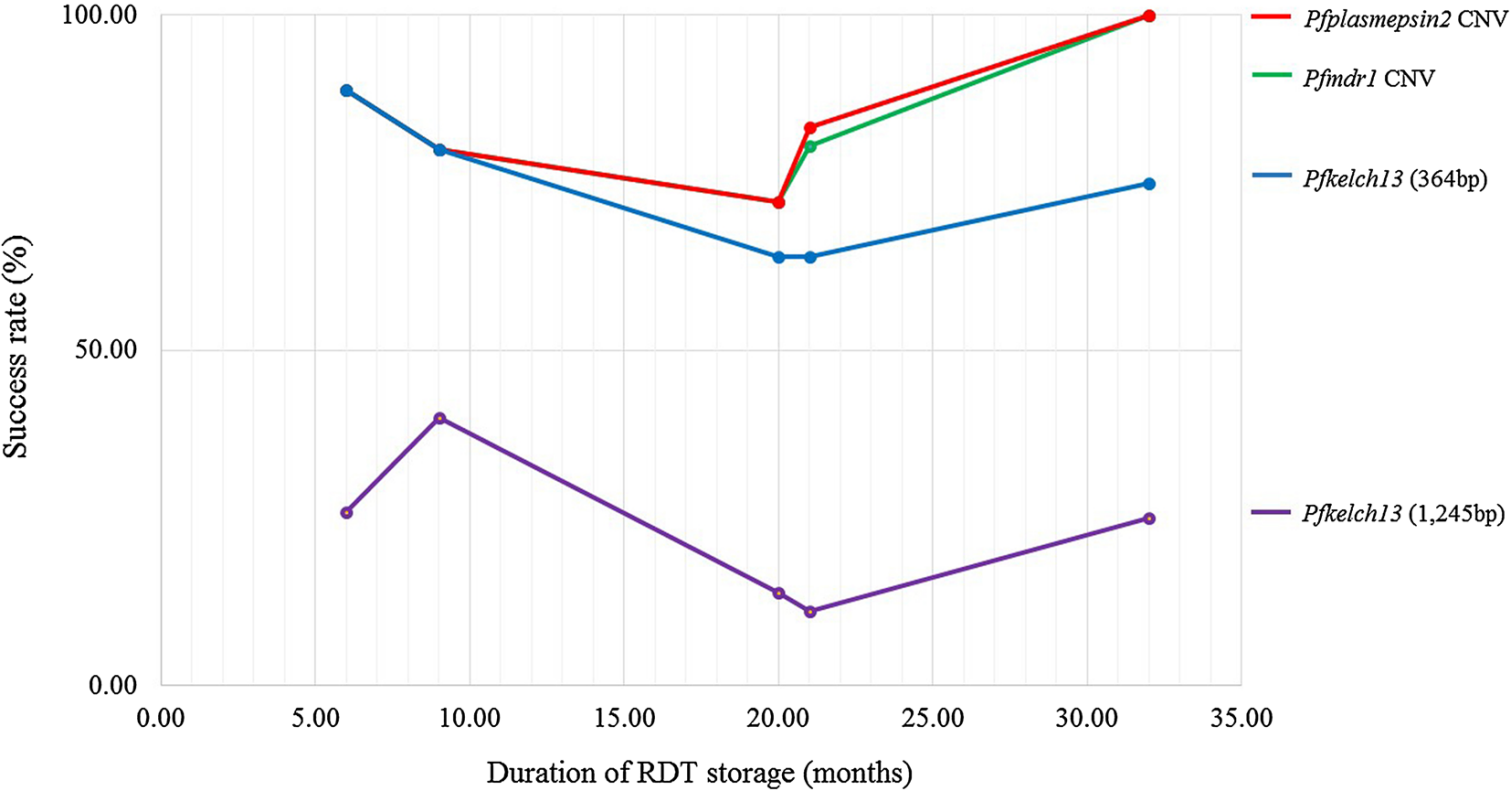
Success rate of molecular analysis using DNA extracted from RDTs

The molecular analysis revealed that: (1) DNA isolated from RDT samples can be successfully used for amplification of *P. falciparum* gene, and no significant difference of success rates exists between storage duration of RDT samples at 6, 9, 20, 21, and 32 months (*p*-*value *> 0.05); (2) Success rate did depend on the molecular marker being assessed: with lower success rate (18%) for long fragment of *pfkelch13* (1245 bp) compared to higher success rate (71.3%) for short fragment of *pfkelch13* (364 bp) (*p*-*value *< 0.05, Chi Square); moreover, the success rates for either *pfmdr1* fragment (87 bp) or *pfplasmepsin2* fragment (108 bp) was 81% (Fig. 2).

### Success of whole genome amplification of *P. falciparum* DNA preps isolated from RDT samples

The total DNA quantity of original DNA extracted from RDT samples was low (1.05 ng/µl on average). After WGA using REPLI-g^®^, MALBAC^TM^, PicoPLEX^®^, and GenomePlex^®^ kits, DNA concentrations increased to 415 ng/µl, 15.30 ng/µl, 32.35 ng/µl, and 2.85 ng/µl, respectively (Table 1). The fold-change in DNA concentration was calculated using the formula:

$$ \frac{{\text{DNA}\,\text{concentration}\,\text{after}\,\text{WGA}\,\left( {{ng/\mu l}} \right)}}{{\text{DNA}\,\text{concentration}\,\,\text{of}\,\text{original}\,\,\text{DNA}\,\left( {{ng/\mu l}} \right)}}. $$

**Table 1:** Descriptive statistics of DNA quantity and quality for original DNA and post-WGA samples amplified by REPLI-g^®^, MALBAC^TM^, PicoPLEX^®^, and GenomePlex^®^ kits

	Descriptive statistics, Mean (CV)				
	Original DNA	REPLI-g^®^	MALBAC^TM^	PicoPLEX^®^	GenomePlex^®^
DNA concentration (ng/µl)	1.05 (± 0.73)	415.00 (± 0.22)	15.30 (± 0.20)	32.35 (± 0.14)	2.85 (± 1.14)
Fold-change of DNA concentration		745.00 (± 1.09)	30.51 (± 1.14)	60.31 (± 1.11)	3.64 (± 1.04)
Estimated parasites DNA quantity (genome/µl)	7789.16 (± 1.90)	66261.49 (± 2.41)	374310.95 (± 1.80)	2095.4563 (± 1.18)	1052.71 (± 1.08)
Fold-change of parasites DNA quantity		745.00 (± 1.09)	30.51 (± 1.14)	60.31 (± 1.11)	3.64 (± 1.04)
Nanodrop (260/280)	0.97 (± 0.29)	1.86 (± 0.01)	1.88 (± 0.02)	1.80 (± 0.01)	1.44 (± 0.11)
Ratio (*P. falciparum*(genome/µl)/Total DNA(ng/µl))	8123.64 (± 1.40)	124.11 (± 2.33)	21,924.46 (± 1.85)	63.37 (± 1.21)	732.43 (± 1.38)

The quality of pre-WGA and post-WGA samples were determined using Nanodrop™. The ratio of absorbance at 260/280 was used to assess DNA purity. Data are represented as Mean (± CV)

**Fig. 3:**
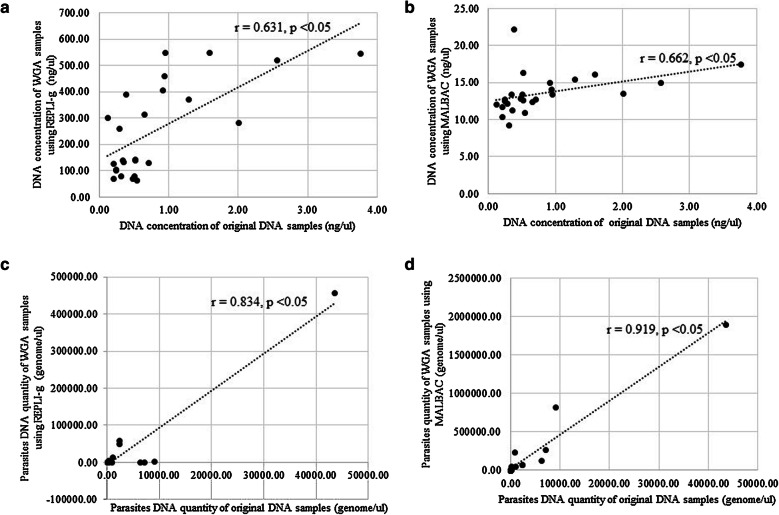
Relationship between DNA concentration of original DNA samples and post-WGA samples using **a** REPLI-g^®^ (n = 25) and **b** MALBAC^TM^ (n = 25). The relationship between parasites DNA quantity estimated by real-time PCR of original DNA samples and post-WGA samples using **c** REPLI-g^®^ (n = 23) and **d** MALBAC^TM^ (n = 24)

The average fold-change in DNA concentrations using REPLI-g^®^, MALBAC^TM^, PicoPLEX^®^, and GenomePlex^®^ were 745.00, 30.51, 60.31 and 3.64, respectively (*p*-*value *< 0.05 for all kits, except for GenomePlex^®^: *p*-*value *= 0.167; Table 1). REPLI-g^®^ provided the highest fold increase in DNA concentrations (Additional file 1).Quantitative real-time PCR estimates of *P. falciparum* DNA (genome/µl) showed a significant increase in parasite DNA quantity between original DNA and post-WGA samples using MALBAC^TM^ kit (Table 1). In contrast, no significant differences were observed using REPLI-g^®^, PicoPLEX^®^, and GenomePlex^®^ (0.167 ≤ *p*-*value *≤ 0.401) (Table 1). *18S rRNA* gene amplification results showed that all four WGA kits could amplify parasite DNA but MALBAC^TM^ yielded the highest fold-change in parasite DNA. The reproducibility expressed as coefficient of variation in WGA yield using the REPLI-g^®^, MALBAC^TM^, PicoPLEX^®^ and GenomePlex^®^ kits ranged from 1.04 to1.14 (Table 1). The ratio of *P. falciparum* DNA quantity (genome/µl) to total DNA (ng/µl) before and after WGA decreased significantly when using REPLI-g^®^, PicoPLEX^®^, and GenomePlex^®^ (Related-Samples Wilcoxon Signed Rank Test, *p*-*value* ≤ 0.05), but increased significantly using MALBAC^TM^ (Related-Samples Wilcoxon Signed Rank Test, *p*-*value* ≤ 0.05) (Table 1). There was a positive correlation between DNA quantity of original DNA and post-WGA using REPLI-g^®^ (*rs *=* 0.631, p*-*value *<* 0.05)* and MALBAC^TM^ (*rs* = 0.662, *p*-*value *<* 0.05)* (Fig. 3). There was also a correlation between the estimated parasites DNA quantity for original DNA samples and post-WGA using REPLI-g^®^ (*rs *= *0.834, p*-*value *<* 0.05*^®^) and MALBAC^TM^ (*rs *=* 0.919, p*-*value *<* 0.05*) (Fig. 3).

### Success of assessment on the quality of *P. falciparum* DNA preps isolated from RDT samples using molecular marker typing

The quality of WGA samples using REPLI-g^®^ and MALBAC^TM^ were assessed regarding accuracy for molecular marker typing. These kits were selected since REPLI-g^®^ yielded the highest fold-change in DNA concentration and MALBAC^TM^ yielded the highest fold-change in estimated parasites concentration.

WGA samples amplified using REPLI-g^®^ (n = 25) and MALBAC^TM^ (n = 25) were used for molecular markers typing, including three real-time PCR assays, four nested PCR assays, and six microsatellite markers. The success rates of molecular marker analysis using REPLI-g^®^ and MALBAC^TM^ kits ranged from 0–100% and 7.69–100%, respectively. There was an association between the success of molecular marker typing and the initial parasites DNA concentrations, both with REPLI-g^®^ (*rs *=* 0.898, p*-*value *<* 0.05*) and MALBAC^TM^ (*rs *=* 0.929, p*-*value *<* 0.05*).

With both kits, *pfmdr1* and *pfplasmepsin2* CNVs was successfully assessed from post-WGA samples in the majority of samples. Concordance with the assessment from original DNA using REPLI-g^®^ kit was high (κ = 1) for *pfmdr1* CNV (n = 15) assessment, but lower (κ = 0.582) for *pfplasmepsin2* CNVs (n = 14). With the MALBAC^TM^ kit (n = 17), concordance for *pfmdr1* CNVs (n = 16) and *pfplasmepsin2* CNVs (n = 17) assessments was low (κ = 0.776 and κ = 0.605, respectively) (Additional file 2). Amplification of *pfkelch13*, *pfdhfr*, *pfcrt*, and microsatellite markers was successful in 32-72% of WGA samples with either REPLI-g^®^ or MALBAC^TM^ WGA kit. The genotyping was accurate in all samples where the target gene was successfully amplified (κ = 1.000).

Cost and time consumption of four WGA kits, namely REPLI-g^®^, MALBAC^TM^, PicoPLEX^®^ and GenomePlex^®^, were estimated and summarized. Performance of MALBAC^TM^ WGA kit was relatively low time-consuming and less labour intensive (Table 2).

**Table 2:** Estimated cost and time consumption of four WGA kits

	REPLI-g^®^(Qiagen)	MALBAC^TM^ (Yikon Genomic)	PicoPLEX^®^ (TaKaRa)	GenomePlex^®^ (Sigma)
Labor intensive	Less	Relative less	More	More
Time consuming^a^	~ 20 h	~ 4 h	~ 4 h	~ 6 h
Cost/sample (USD)^a^	18.73	31.92	37.11	12.90

^a^The cost and time consumption may subjected to vary depending upon laboratory facilities and taxes

## Discussion

Used RDTs provide a valuable source of parasite DNA that can be used for molecular analysis [2–4, 7, 8]. The current study investigated the utility of DNA preps from RDT samples for *pfkelch13* SNPs, *pfmdr1* CNV and *pfplasmepsin2* CNV analysis. Even though theDNA preps were of limited quantity and with degradation, molecular typing was successful in 17.59–81.48% of samples. Storage up to 32 months did not negatively affect the accuracy of typing, showing that stored RDTs can be used as a source for *Plasmodium* DNA typing as also suggested in earlier studies [2, 4, 7, 8]. Comparing short (364 bp) and long (1,245 bp) fragment *Pfkelch13* amplicons suggested that PCR product size affects the success rate of molecular analysis using RDT blood spots.

Inadequate amounts of parasite DNA is a limiting factor for conducting molecular assays from RDT blood spots of around 5 µl, in particular when several markers are evaluated [25, 26]. RDT samples were collected from symptomatic cases from the area where the observed geometric mean *P. falciparum* parasitaemia was reported as 6 *Plasmodium* genome equivalents/µL (95% CI 2 to 16), so around 30 (95% CI 10 to 80) genomes per blood spot [27]. The purity and amount of DNA will affect outcomes of downstream molecular analysis. Although the WGA using any of the four kits increased parasite DNA, only WGA with REPLI-g^®^ and MALBAC^TM^ yielded sufficient *P. falciparum* DNA purity and quantity needed for further molecular analysis. MALBAC^TM^ yielded higher concentrations of parasite DNA compared to REPLI-g^®^, which makes this kit preferable for amplification using RDT blood spots with low parasites content. A high concordance in microsatellite marker and SNP typing was previously reported using the MDA^®^ kit to amplify parasite DNA from whole blood samples of *P. falciparum*-infected patients [13]. The current study shows reliable results for molecular marker typing, DNA sequencing and microsatellite marker analysis using REPLI-g^®^ and MALBAC^TM^ WGA of RDT blood spots. However, CNV estimates for *pfmdr1* and *Pfplasmepsin2* were less reliable, suggesting that WGA amplified DNA should not be used for the purpose of CNV assessment. A previous study showed that PicoPLEX^®^ and MALBAC^TM^ provided reliable CNVs estimates based on an ion proton platform, whereas CNV estimates were not accurate using MDA or GenomePlex^®^ [16]. As expected from the working principles of WGA and confirmed by the current study, WGA interferes with the assessment of CNV in the parasite genome. This study showed that the success rate of molecular marker analysis was affected by the initial parasite DNA concentration, and WGA with MALBAC^TM^ achieved a higher success rate for molecular marker typing than REPLI-g^®^ in concordance. Although an estimated cost of MALBAC^TM^ is higher than REPLI-g^®^, the use of MALBAC^TM^ was less time consuming compared to REPLI-g^®^ and provided higher yield of parasite DNA. Therefore, MALBAC^TM^ shall be recommended for wide use to generate high-quality parasite DNA in sufficient quantities using stored RDT blood spots containing low parasite DNA quantities. Undoubtedly, good laboratory practices and good laboratory facilities are important to implement these techniques.

## Conclusions

This study demonstrates the successful utility of RDT blood spots for molecular analysis and whole genome amplification. WGA from RDT blood spots using the MALBAC^TM^ kit yields high-quality parasite DNA in sufficient quantities for preforming molecular assays.

## Supplementary information

**Additional file 1.** Bar Chart of DNA concentrations comparing original DNA and post WGA samples using four WGA kits: REPLI-g^®^, MALBAC^TM^, PicoPLEX^®^, and GenomePlex^®^.

**Additional file 2.** Analytical concordance (based on kappa statistic) of *Pfmdr1* CNVs and *Pfplasmepsin2* CNVs results obtained from original DNA samples extracted from RDT and post-WGA samples using REPLI-g^®^ and MALBAC^TM^ kits.

## Data Availability

All data generated or analysed during this study are included in this published article and its supplementary information files.

## Abbreviations

DNA: Deoxyribonucleic acid
RDTs: Malaria rapid diagnostic tests
WGA: Whole genome amplification
*18S rRNA*: 18s ribosomal RNA gene
*Pfmdr1*: *P. falciparum* multidrug resistance 1 gene
*Pfplasmepsin2*: *P. falciparum* plasmepsin2 gene
*Pfkelch13*: *P. falciparum* kelch 13 gene
*Pfcrt*: *P. falciparum* chloroquine resistance transporter gene
*Pfdhfr*: *P. falciparum* dihydrofolate reductase gene
bp: Base pair
ng: Nanogram
µl: Microlitre
