# Supplementary material for: Utility of Plasmodium falciparum DNA from rapid diagnostic test kits for molecular analysis and whole genome amplification
Source: Malar J. 2020 May 27;19:193. doi: 10.1186/s12936-020-03259-9 (PMC7251736; doi:10.1186/s12936-020-03259-9)
Supplement: Supplementary file 1 — Additional file 1. Bar Chart of DNA concentrations comparing original DNA and post WGA samples using four WGA kits: REPLI-g®, MALBACTM, PicoPLEX®, and GenomePlex®. [file 12936_2020_3259_MOESM1_ESM.pdf]

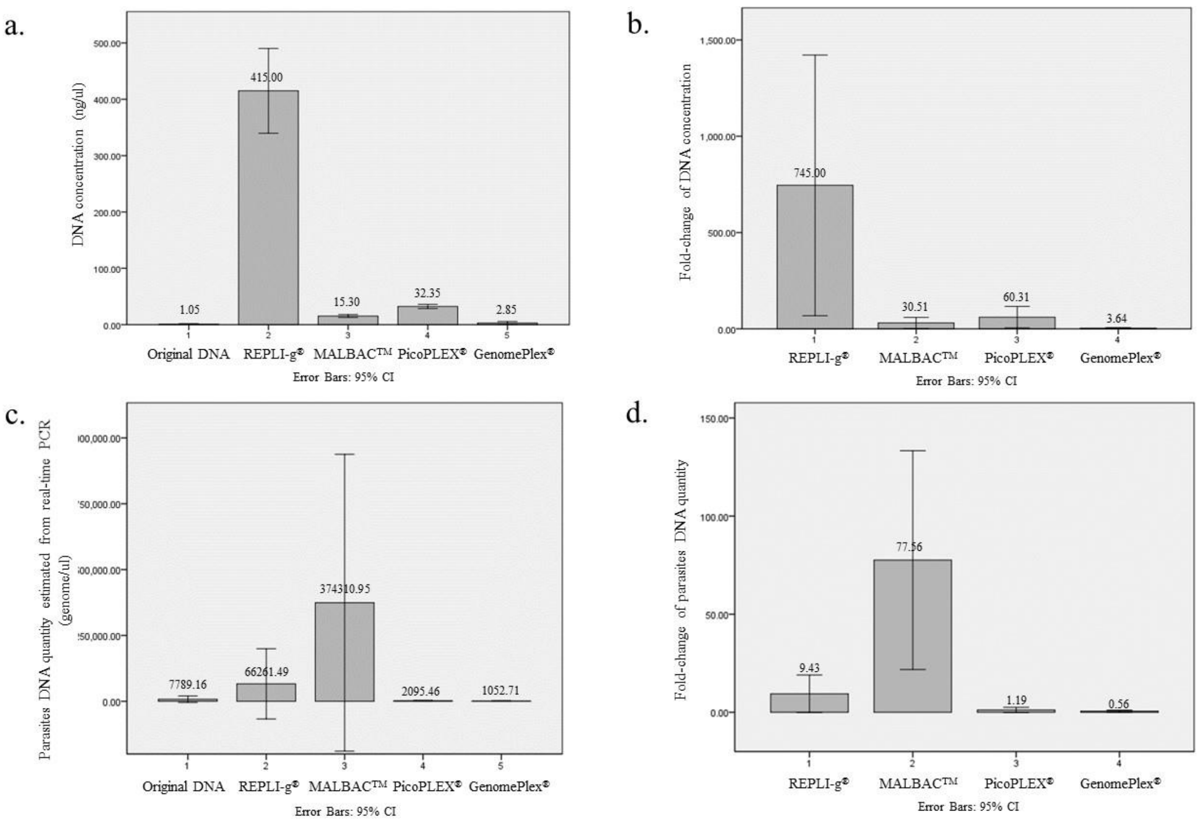


**Additional file 1.** Bar Chart of DNA Concentration of original DNA and WGA samples using four WGA kits, REPLI-g®, MALBAC™, PicoPLEX®, and GenomePlex® kits(a), fold-change of DNA concentration using four WGA kits (b), parasite DNA quantity estimated from real-time PCR (c), and fold-change of parasite DNA quantity using four WGA kits (d).
