# Supplementary material for: Utility of Plasmodium falciparum DNA from rapid diagnostic test kits for molecular analysis and whole genome amplification
Source: Malar J. 2020 May 27;19:193. doi: 10.1186/s12936-020-03259-9 (PMC7251736; doi:10.1186/s12936-020-03259-9)
Supplement: Supplementary file 2 — Additional file 2. Analytical concordance (based on kappa statistic) of Pfmdr1 CNVs and Pfplasmepsin2 CNVs results obtained from original DNA samples extracted from RDT and post-WGA samples using REPLI-g® and MALBACTM kits. [file 12936_2020_3259_MOESM2_ESM.pdf]

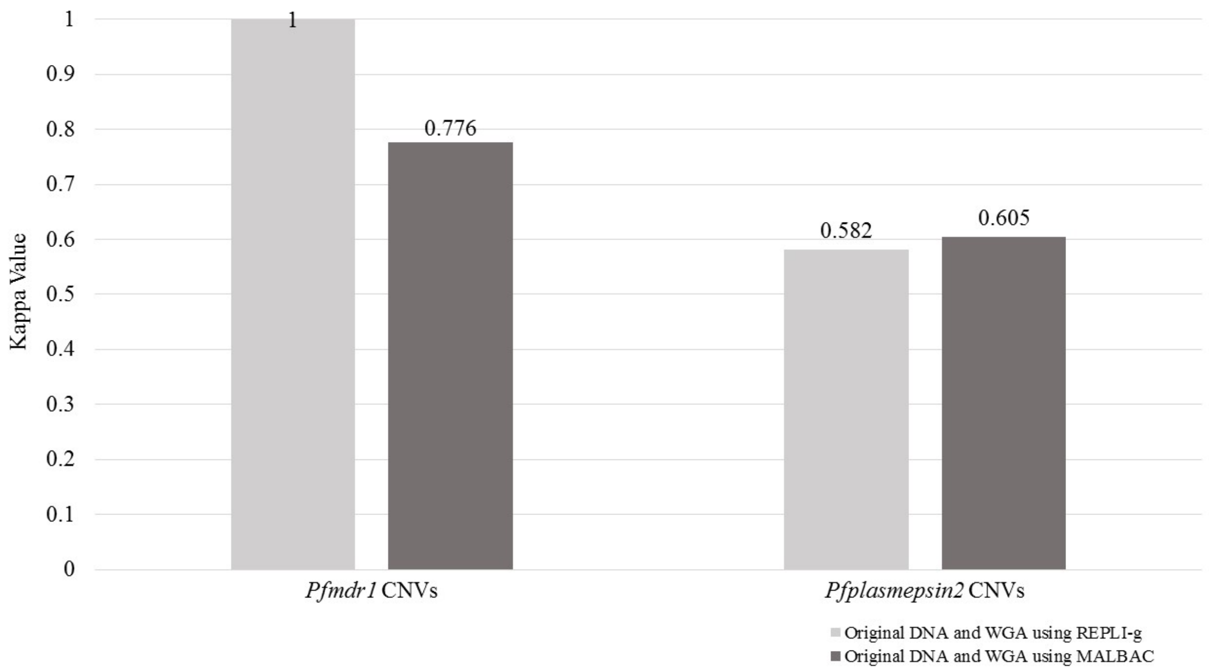

Additional file 2. Analytical concordance (based on kappa statistic) of *Pfmdr1* CNVs and *Pfplasmepsin2* CNVs results obtained from original DNA samples extracted from RDT and post-WGA samples using REPLI-g® and MALBAC™ kits.
